# Development of Eco-Friendly Date Palm Biomass-Based Hydrogels for Enhanced Water Retention in Soil

**DOI:** 10.3390/gels11050349

**Published:** 2025-05-08

**Authors:** Faisal S. Alsubaie, Mouyed Srdar, Osama Fayraa, Faris M. Alsulami, Feras Omran, Khalid A. Alamry

**Affiliations:** Chemistry Department, Faculty of Science, King Abdulaziz University, Jeddah 21589, Saudi Arabia; mabdoalhaqserdar@stu.kau.edu.sa (M.S.); oifayraa@stu.kau.edu.sa (O.F.); fbaderalsulami@stu.kau.edu.sa (F.M.A.); fmohammedomran@stu.kau.edu.sa (F.O.)

**Keywords:** hydrogel, lignocellulosic biopolymers, all-lignocellulose, water retention, soil conditioning, germination, plant growth

## Abstract

The growth of plants highly depends on the soil’s water availability and properties. Hydrogels (HGs) have been used for decades to enhance soil water retention, whereas developing eco-friendly and sustainable HGs for agricultural applications is still necessary to ensure water and food security. In this study, renewable and cost-effective HGs were prepared from all-lignocellulose fibers of date palm biomass after carboxymethylation followed by citric acid (CA) crosslinking. HGs showed high equilibrium swelling capacity (EWC%), even in salty media, whereas purified HGs showed about 700–400 EWC% in deionized water. Further, HGs’ effect on germination was studied on Chico III tomato, mint, Basilico red, and chia seeds. The results revealed that HGs enhanced the soil properties, with taller and healthier plants observed in HG-amended soil. FTIR, thermal analysis, and microscope imaging were utilized to evaluate HGs’ and raw materials’ characteristics. The findings in this study support the idea that all-lignocellulose could be used for HG production without separation.

## 1. Introduction

Sustainable agriculture systems, water availability, and proper soil are key to food security. The agriculture sector in arid and semi-arid regions faces multiple challenges due to drought, desertification, climate change, sandy soil, and harsh weather. In arid conditions, irrigational water tends to evaporate from the soil due to high temperatures. Also, the soil type stimulates water infiltration, increasing irrigational water demand and irrigation cycles. Further, arid and semi-arid regions suffer from a shortage of freshwater resources [1,2,3,4]. On the other hand, the demand for freshwater is increasing daily due to the growing population, urbanization, and industrialization. By 2030, the expected water demand will be 50% higher than today’s demand. This may result in water scarcity if efficient water management systems are still underdeveloped [5]. It is worth mentioning that about 70% of global freshwater was estimated to be used for the irrigation of only 25% of the world’s croplands which supply only 45% of international food [6]. A helpful solution may lie in inventing sustainable irrigation systems and soil conditioners for water preservation to reduce the total water demand from the farming sector [5]. In 2021, Saudi Arabia initiated the “Saudi & Middle East Green Initiatives” to plant more than 10 billion trees to face climate change, desertification, and air pollution [7]. This number of trees will require vast irrigational water, arable soil, and rehabilitated land, which are mandatory for these initiatives to succeed, considering that water resources are scarce in regions like the Middle East and North Africa (MENA). Hence, local, modern solutions to enhance planting should be explored and utilized.

Hydrogels (HGs) and superabsorbent hydrogels (SHs), also known as superabsorbent polymers (SAPs), have been an attractive solution for soil conditioning since the 80s owing to their moisture and water preservation ability [8]. HGs are formulated from different types of crosslinked hydrophilic polymers. Different physical and chemical methods could be used for crosslinking polymer chains. The physical crosslinking methods lean on physical interactions like hydrogen bonding and Van der Waals interactions, while chemical crosslinking relies on covalent bonds formation. Epichlorohydrin, glutaraldehyde, and citric acid (CA) are some of the commonly used crosslinking agents [3]. CA is considered an eco-friendly and cost-effective crosslinker in comparison with other common crosslinkers like epichlorohydrin and glutaraldehyde [9]. After crosslinking, HG materials can hold water, organic matter, or inorganic materials within their 3D network matrix and release them upon some stimulus, such as pH, temperature, and ionic strength [3,10,11]. Investigations have found that HGs have huge water capacities comprised of their original weight, which could be higher than 1000% of their weights, enhancing soil moisture and oxygenation [12]. HGs have been well-utilized in the industrial field in various applications, such as in the biomedical and agricultural industries. Many studies have explored HGs and their effects as a soil conditioner, and they have been considered an adequate solution for enhancing planting and soil [13,14]. Demitri et al. studied the impact of HGs prepared from hydroxyethylcellulose (HEC)/carboxymethylcellulose sodium salt (CMCNa) that was citric acid-crosslinked on cherry tomato cultivation in greenhouses and claimed that HG-amended soil showed three times higher humidity with respect to the control soil. Also, longer survival times were observed in hydrogel-containing soil [15]. Chitosan (CS) is a chitin-derived polymer that is highly abundant in living organisms and has also been utilized for hydrogel production and for controlled fertilizer release. Jamnongkan et al. developed chitosan/poly(vinyl alcohol)-based hydrogel for the controlled release of potassium. Their results revealed that the incorporation of hydrogel in the soil enhanced the water retention period [16]. In addition, starch-based HGs have been applied in many studies for agricultural applications. Starch is one of the most abundant polysaccharides, and it is biodegradable and biocompatible. Chamorro et al. developed citric acid-crosslinked cassava starch hydrogels for the slow release of ammonium. They claimed that the prepared hydrogel reduced the fertilizer release rate in the soil [9]. Both synthetic polymers and natural polymers have been used for HG preparation by physical or chemical crosslinking. To some extent, synthetic HGs have some drawbacks regarding their environmental footprint. Therefore, natural polymers are a good candidate due to their biodegradability, biocompatibility, and natural abundance [12,17]. Natural polymers or biopolymers are the types of polymers that can be found in nature within biological systems, whether in plant-based or animal-based systems.

Agricultural biomass is one of the wealthiest sources of biopolymers. It is a plant-based agrarian waste that is biodegradable, biocompatible, and highly available. Biomass is an underutilized mine of biopolymers, and it mostly becomes landfill waste or is burned, which is considered polluting and a waste of valuable resources. The lignocellulosic matrix is the main constituent of biomass, comprising about 65% of biomass weight. This matrix comprises polysaccharides, such as cellulose and hemicellulose, alongside lignin, a polyphenol biopolymer [18,19]. These biopolymers have been utilized for decades directly or after modification toward different applications and uses. Cellulose is the main component of the lignocellulose matrix, and it is the most abundant natural polymer on earth. Cellulose is a homopolymer of anhydroglucoses (C_6_H_10_O_5_) linked by β-1,4 glycosidic bond, and the result is a water-insoluble and highly crystalline long-chain polymer. The second major component is hemicellulose, which is amorphous in structure and constituted of different penta- and hexa-sugars. The third component by percentage is lignin, which is composed of three different phenols with alternative ratios, which are p-coumaroyl alcohol, coniferyl alcohol, and sinapyl alcohol, forming three-dimensional polyphenol [20,21]. Many studies have used those biopolymers to prepare hydrogels and show their capability in different applications [22,23,24]. Several pretreatments are required to reduce the biomass recalcitrance and separate the biopolymers from the matrix to produce HGs. Common pretreatments for biomass recalcitrance reduction are costly and energy extensive [25]. The most efficient and common pretreatments are chemical pretreatments, but unfortunately, they include the use of harsh chemicals that are accompanied by environmental pollution [26,27]. Also, it is worth mentioning that lignocellulosic biopolymers are naturally insoluble in water, so chemical modification reactions should be performed to yield water-soluble polymers to form HGs. Many chemical modifications, including acetylation [28] and etherification [29], have been applied to biopolymers to enhance their solubility and reactivity. To increase water solubility, carboxymethylation is considered one of the most common modifications for polysaccharides, where a carboxymethyl group is introduced to the biopolymer molecular chain in an etherification reaction with monochloroacetic acid (MCA), which is a well-studied reaction [30].

The date palm tree (*Phoenix dactylifera* L.) is one of the most common crops cultivated in the MENA and is one of the oldest tree species. Worldwide, there are approximately 100 million date palm trees [31]. In Saudi Arabia alone, there are about 30 million date palm trees, where the present research was conducted [32]. In addition, there are about 40 million trees in the United Arab Emirates (UAE) [28], 18.7 million in Algeria [29], and 4.45 million in Morocco [31]. In Saudi Arabia, the amount of date palm biomass released is about 1 million metric tons annually, and it is primarily underutilized and disposed of in landfills or burned [32]. In addition, date palm biomass, specifically date palm rachis (DPR), is one of the most common non-edible date byproducts and is full of a lignocellulosic matrix, which can be fractionated and used as biopolymers or used according to the desired application [33]. It is worth mentioning that, like other biomass, date palm biomass contains 60–95% biopolymers [34].

This study aims to utilize DPR biomass to prepare HGs for water retention in soil using a facile and eco-friendly method. The suggested approach is to directly convert DPR to carboxymethyl date palm rachis (CMDPR) by the MCA etherification reaction without chemically pretreating the biomass before the modification reaction, reducing the production cost and footprint. The prepared water-soluble product was crosslinked by an eco-friendly agent, i.e., CA, using different ratios to yield HGs. Different tests and characterization were applied to the resulting CMDPR to confirm the modification occurrence and to the prepared HGs to confirm their suitability in soil water retention. The prepared HGs showed suitable water swelling, thermal stability, and a positive impact on plant germination.

## 2. Results and Discussion

### 2.1. Chemical Composition of DPR and Pretreatment

The chemical composition of DPR is shown in Table 1. The DPR used in this study contained 13.74%, 29.82%, and 21.69% of lignin, α-cellulose, and hemicellulose, respectively, which is similar to the ratios that are found in the literature and presented in Table 1 [31,35,36,37]. Based on Table 1, cellulose content varied between 47–29% of DPR weight. The hemicellulose and lignin also varied in different collected DPR from different places in a lower range. These variations were mostly due to differences between soil conditions, geographic location, and climate [38]. As a result, the total amount of lignocellulosic biopolymer (lignin, cellulose, and hemicellulose) within the DPR used in this study was 65.25% of the total weight of the DPR. Total lignocellulosic biopolymers indicate the total of the most modifiable polymers (that could be carboxymethylated). Two types of pretreatments were performed on DPR to ensure efficient carboxymethylation: physical pretreatment, which includes grinding and milling, followed by screening. This pretreatment was performed to increase the modification degree, where the particle size tremendously impacts the degree of substitution [39]. Subsequently, hot water pretreatment was performed to reduce the amount of hot-water-soluble (HWS) matter to avoid the possibility of reaction reagents consumption, i.e., NaOH and MCA, during the modification reaction [40].

### 2.2. Characterization of DPR and WTDPR

**Figure 1:**
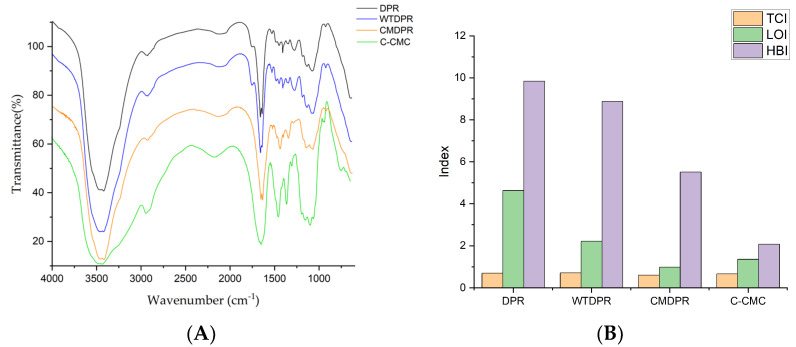
FTIR spectrum (**A**) and crystallinity indices (**B**) of DPR, WTDPR, CMDPR, and C-CMC.

The pretreated DPR was named water-treated DPR (WTDPR), which was then characterized by comparison with DPR. Fourier-transformed infrared spectrometry (FTIR) was employed to confirm the structural differences and crystalline indexes, while morphological characteristics were evaluated by a digital microscope and dynamic light scattering (DLS). The FTIR spectrum DPR and WTDPR are shown in Figure 1A. Both samples presented typical vibration bands of lignocellulosic biopolymers, i.e., cellulose, hemicellulose, and lignin chemical functional groups, as presented in Table 2. A broad absorption band was observed around 3400 cm^−1^, which is attributed to the −OH group stretching of lignocellulose biopolymers and water, whereas the absorbance band at 2919 cm^−1^ was assigned to the −CH group stretching vibrations from cellulose and hemicellulose [41]. A peak observed at 1731 cm^−1^ was attributed to C=O of ester or carboxylic acid group in hemicellulose and lignin [42,43], and a vibration band was observed at 1637 cm^−1^, which was attributed to conjugated carbonyl group [43]. In the following, three sequential peaks were attributed to the lignin aromatic ring at 1618 cm^−1^, which was assigned aromatic skeletal vibration of lignin, and −C=O stretching of the lignin ring plus flavonoids [44,45]; a second peak at 1507 cm^−1^, which was assigned to −C=C stretching of the lignin ring; and lastly at 1458 cm^−1^, which was assigned to −CH group deformation within the lignin ring [46,47]. The absorbance at 1425 cm^−1^ was mainly attributed to the −CH_2_ symmetric bending in cellulose and −CH deformation in the lignin ring [47]. The absorption band at 1374 cm^−1^ was assigned to the bending vibration of the −CH group of hemicellulose and cellulose, while the band at 1321 cm^−1^ was attributed to the −CH_2_ rocking vibration in cellulose [47]. The peak at 1250 cm^−1^ was assigned to the C−O stretching of the acetyl group in the lignin [48]. The peak at 1158 cm^−1^ was assigned to C−O−C asymmetric stretching in hemicellulose and cellulose or the ester group in lignin, while the peak at 1049 cm^−1^ was attributed to ether group C−O stretching vibration within the cellulose, hemicellulose, and lignin [46,47]. The band at 1108 cm^−1^ was attributed to C−C/C−O stretching within the aliphatic skeletal of cellulose and hemicellulose [49]. The band at 897 cm^−1^ was assigned to the β-glycosidic bonds between the monosaccharides [50]. It is noteworthy that there were no notable peak absences since the hot water treatment does not affect cellulose and lignin structure while negligibly affecting hemicellulose [51].

Different indices were calculated utilizing the ratio of certain bands’ intensities from the FTIR spectra of samples to evaluate the crystallinity changes. The lateral order index (LOI) is an empirical crystallinity index of cellulose correlated to the overall order degree of cellulose. LOI was proposed by Nelson and O’Connor, alongside the total crystallinity index (TCI), which is proportional to the crystallinity degree of cellulose [52,53]. In addition, the hydrogen bond intensity (HBI) index was also used to evaluate the crystallinity of samples, which was proposed by Nada et al. [54]. HBI also indicates the degree of crystallinity and bound water content, where it increases as crystallinity decreases. By calculating the ratio between the absorbance intensities of CH_2_ flexion at 1430–1420 cm^−1^ of the crystalline cellulose and the intensity of the amorphous cellulose at 900–890 cm^−1^ that is assigned to the β-glycosidic bonds between the monosaccharides, LOI could be obtained [55,56]. TCI is the ratio between the absorbance intensities at ~2900 cm^−1^, which corresponds to stretching vibrations of –CH, and ~1374 cm^−1^, which is assigned to the deformation vibration of CH in cellulose. In contrast, TCI is proportional to the degree of crystallinity of cellulose [55,57]. On the other hand, HBI is the ratio between the absorbance intensities of OH stretching and hydrogen bonding between molecules around 3330–3400 cm^−1^ and the absorbance of at ~1320 cm^−1^ from CH rocking vibration of glucose ring [58,59]. The TCI was obtained from the intensity ratio of A1374/A2919 and LOI from the ratio A1425/A897. The ratio A3336/A1321 was used for HBI. The TCI of DPR was found to be 0.697 with 3.245 LOI and 3.647 HBI, as presented in Figure 1B. After the hot water treatment, both TCI and LOI were noticed to increase to 0.707 and 3.635, respectively. The resulting increase was mostly attributed to the reduction in HWS and extractives, where samples with higher extractive content showed less TCI [59]. Furthermore, the HBI was found to decrease to 3.005 [59]. These results are in harmony with findings in the literature regarding the hot water treatment effect on biomass, where researchers have found that both LOI and TCI increased with liquid hot water treatment in different ranges of temperature and pretreatment time [60]. Also, another study found that LOI and TCI increased after thermal pretreatment from 1.346 to 1.384 and from 0.282 to 0.295, respectively, when the temperature increased from 20 °C to 160 °C [61]. In addition, researchers have found that TCI increases by increasing the sterilization temperature with a reduction in both LOI and HBI; this may be due to the degradation of hemicellulose and amorphous fraction of cellulose, which results in cellulose with better crystallinity [62].

**Figure 2:**
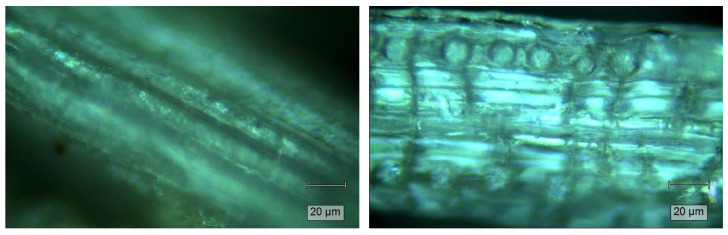
Digital microscope imaging of DPR (**left**) and WTDPR (**right**).

Figure 2 shows the morphology of DPR and WTDPR fibers, which was recorded by a digital microscope. The DPR fiber shows a smooth surface with a compact structure and aligned ordered microfibril lines. Morphological changes were observed after the hot water treatment, where the WTDPR fiber showed a swelled structure with a rough surface and some cracks. Additionally, small holes are observed after the hot water treatment, which may attributed to the leaching of silica bodies from WTDPR. These results are similar to biomass after different pretreatments, where cracks and silica cavities were observed [50,63].

**Figure 3:**
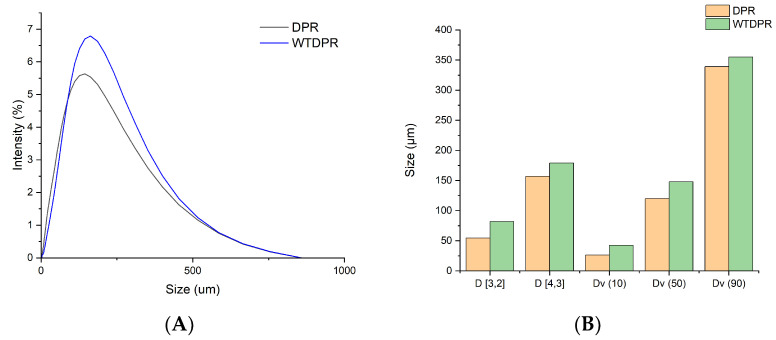
Size distribution of particles by intensities (**A**) diameter and distribution by size distribution (**B**).

The results of the DLS analysis of DPR and WTDPR are shown in Figure 3. The analysis was performed to assess the particle size distribution change after the milling and hot water treatment. The volume-weighted mean diameter D_[4,3]_ of DPR was 157 µm. The median volume distribution of the Dv_(50)_ particles was 120 µm, meaning that 50% of the particles were smaller while 50% were larger. After the hot water treatment, an increase was recorded where D_[4,3]_ was 179 µm while recorded Dv_(50)_ was 148 µm; the uniformity was found to be 0.805 and 0.657 for DPR and WTDPR, respectively.

This may have been due to the removal of hot water solubles and silica, which increased the accessible surface area of the lignocellulosic biopolymers. This explanation is in harmony with findings in the literature, where it was reported that the degradation of hemicellulose or lignin increases the surface area of cellulose, which promotes aggregation [64,65]. Besides, the aqueous media and drying process were reported to promote the self-aggregation of lignocellulose biopolymers [66,67].

### 2.3. Preparation of CMDPR, Degree of Substitution, and Yield

**Figure 4:**
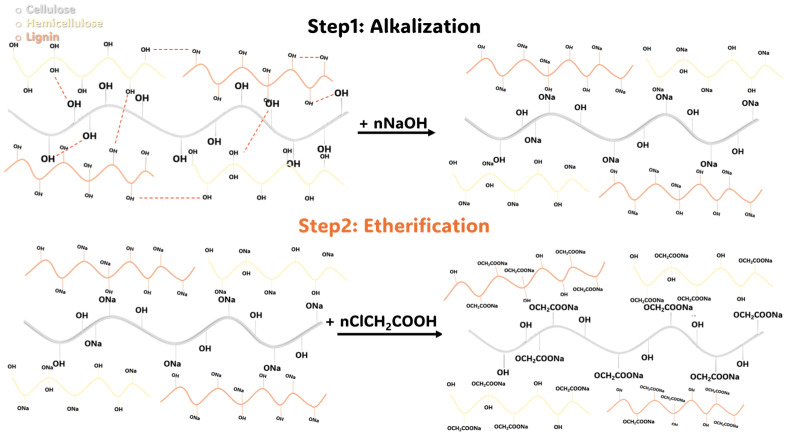
Carboxymethylation reaction of lignocellulosic matrix.

**Figure 5:**
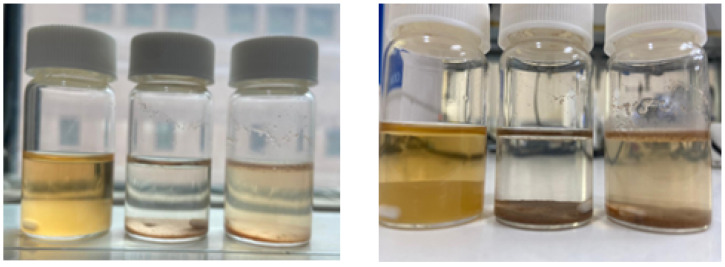
Visual photos of 1% (*w*/*v*) mixtures of CMDPR (**left**), WTDPR (**mid**), and DPR (**right**) after 1 h of stirring.

Carboxymethyl date palm rachis (CMDPR) was prepared from WTDPR with 179 µm D_[4,3]_. The carboxymethylation was carried out according to the known two-step carboxymethylation reaction. In the first step, NaOH activated lignocellulose biopolymers’ hydroxyl groups, and in the second step, MCA reacted with the activated groups to yield CMDPR, as shown in Figure 4. The resulting CMDPR mainly comprised carboxymethyl lignin, carboxymethyl cellulose, and carboxymethyl hemicellulose. The estimated degree of substitution of the resulting CMDPR was found to be 1.14, with high solubility and fine low insoluble particles, as shown in Figure 5, compared with DPR and WTDPR visually. The yield was 137.29%, which is close to the findings in other literature (Table 3). A study reported that using date palm rachis with a size range of 200 µm–1000 µm resulted in carboxymethylated biomass with 1.17 DS [68]. Another study showed that holocellulose with a particle size of 100 resulted in CMC with 1.83 DS and 182.55% yield [39]. Besides the other reaction conditions, particle size has an important effect on DS.

### 2.4. Characterization of CMDPR and C-CMC

The prepared CMDPR was characterized using FTIR, a digital microscope, thermalgravimetric analysis (TGA) in an oxygen-rich atmosphere, and differential scanning calorimetry (DSC) and compared with commercial carboxymethyl cellulose (C-CMC) with 0.8 DS.

FTIR spectroscopy was used to confirm the carboxymethylation of WTDPR and other structural changes regarding the functional groups and crystallinity indices. Significant changes were observed in the FTIR spectrum between WTDPR and CMDPR, whereas a noticeable reduction of the following peaks around 1158 cm^−1^, 1250 cm^−1^, and 1731 cm^−1^ were noticed in CMDPR, as shown in Figure 1A. These peaks were assigned to the ether or esters groups of lignocellulosic polymers, C−O of acetyl of lignin, and ester or carboxylic groups in hemicellulose and lignin, respectively [43,46]. The absence of these peaks indicates the possibility of hemicellulose and lignin solubilization or the hydrolysis of the mentioned functional groups due to the alkalization step, which tends to hydrolyze branched groups on lignin and hemicellulose [71,72,73,74]. An observed reduction in the band at 1374 cm^−1^ also supports the possibility of hemicellulose solubilization, which is assigned to the bending vibration of the −CH group in hemicellulose and cellulose. Intensity increases were noted in three bands associated with carboxymethylated polysaccharides in prepared CMDPR at 1618 cm^−1^, 1425 cm^−1^, 1326 cm^−1^, which were assigned to −C=O stretching in the carboxyl group, the vibration of the carboxyl groups salts, and −OH bending vibration [39]. Those peaks were also observable in the C-CMC with 0.8 DS. It is noteworthy that commercial carboxymethyl cellulose is often prepared from pure cellulose; for this reason, aromatic bands of lignin around 1637 cm^−1^, 1507 cm^−1^, 1458 cm^−1^, and 1374 cm^−1^ were not detected in comparison with CMDPR, which was prepared from a lignocellulosic matrix that contains lignin and hemicellulose.

Furthermore, crystallinity changes occurred within CMDPR (Figure 1B), where TCI decreased from 0.707 in WTDPR to 0.606 in CMDPR. Also, a drastic reduction in LOI was observed from 3.635 in WTDPR to 0.977 in CMDPR. As expected, an increase in HBI was noticed from 3.005 to 3.413, where HBI increased with a decrease in crystallinity. C-CMC showed better TCI at 0.662 and 1.351 LOI and lower HBI at 2.088. These results match with the results from other studies using different techniques, where it was found that the carboxymethylation reaction highly reduced the crystallinity of cellulose, which was most likely due to the substitution of hydroxyl groups with carboxymethyl groups and sodium hydroxide ions, which cleave and restrict the formation of hydrogen bonding, hindering the adoption of order arrangements and increasing the distance between the chains [75,76,77,78]. This may explain the reason that C-CMC with a lower DS of 0.8 had higher crystallinity than CMDPR with a higher DS of 1.14.

**Figure 6:**
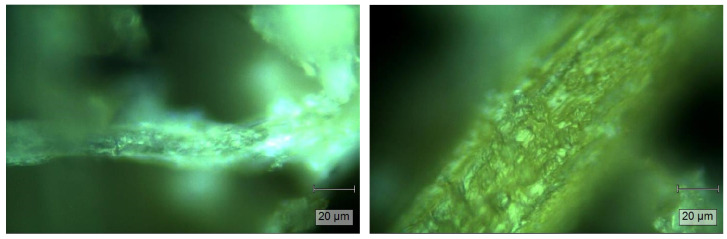
Digital microscope imaging of carboxymethylated date palm rachis (CMDPR).

Figure 6 shows the micrographs of CMDPR, which also confirmed the separation of fibers. Meanwhile, much smaller fibers appeared after carboxymethylation with smooth surfaces, reduced diameters, and ribbon-like shapes compared to WTDPR and raw DPR, which showed relatively compact structures. These results are in alignment with other studies [76,79,80]. In addition, a change in fiber color was observed, where the fiber color turned to golden yellow-like from brown in WTDPR.

**Figure 7:**
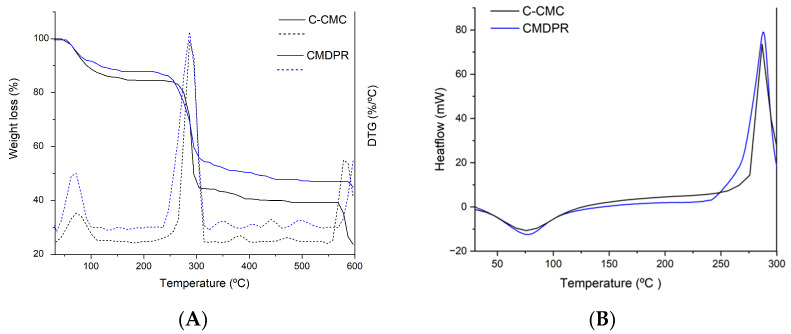
TGA, DTG (**A**), and DSC analyses (**B**) of CMDPR and C-CMC.

TGA and DSC established CMDPR with 1.14 DS and C-CMC with 0.8 DS thermal characteristics and stability. Figure 7A presents TG curves and the derivative DTG curves of both CMDPR and C-CMC, showing decomposition peaks, which are illustrated in Table 4. An initial mass loss started at 50 °C and ended at 180 °C in both samples, which was most likely due to the evaporation of volatile organic and absorbed moisture attributed to the hygroscopic nature of the samples. The initial mass loss percentages were 12.16% for CMDPR and 15.26% for C-CMC. Further, several decomposition peaks were observed in CMDPR between 230 °C and 530 °C. The decompositions in this range mainly comprised the degradation of carboxymethyl groups alongside the degradation of the lignocellulosic matrix. Cellulose, hemicellulose, and lignin degradation were exhibited at 360–400 °C, 200–315 °C, and 160–900 °C [39,81]. In both samples, a typical decomposition curve of carboxylic and hydroxyl groups was observed; the decomposition of CMDPR was between 230 °C and 324 °C, with a weight loss of about 33.5%. For C-CMC, the decomposition started at 246 °C and went to 314 °C with a weight loss of about 39.7% [82]. In addition, a degradation peak was observed between 324 and 373 °C in CMDPR and between 360 and 400 °C in C-CMC, mostly representing the degradation of cellulose [47]. The decomposition at 382–421 °C represents the methyl-aryl ether bonds of lignin, whereas the decomposition between 421 °C and 460 °C was due to the degradation of lignin polymeric backbone chains [83]. The last decomposition peak in both CMDPR and C-CMC was attributed to the oxidation of residual carbonaceous materials [82].

Figure 7B presents the DSC analysis for C-CMC and CMDPR. For both samples, a single endothermic peak attributed to the glass transition (*T_g_*) and a single exothermic peak attributed to the polymer destruction were observed, which were confirmed by TGA. For C-CMC, the *T_g_* was found at 71 °C, while for CMDPR, it was at 75 °C. The polymer destruction for C-CMC was about 286 °C and 288 °C for CMDPR. These results prove that CMDPR has good thermal stability in comparison with C-CMC.

### 2.5. Hydrogels Fabrication and Characterization

**Figure 8:**
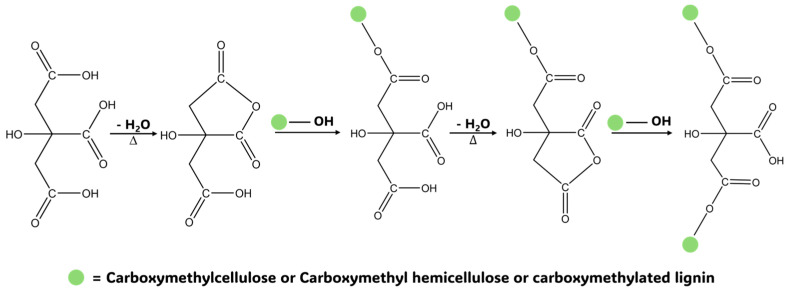
Illustration of citric acid crosslinking reaction mechanism with carboxymethylated biopolymers.

Three different concentrations of CA were chosen to assess the HG preparation potential of prepared CMDPR 10%, 15%, and 20% and the resulting HG-abbreviated CM10, CM15, and CM20, respectively. The gelation mechanism is illustrated in Figure 8. The gelation reaction consisted of two major steps. In contrast, CA underwent a dehydration reaction to yield cyclic anhydride that further reacted with OH groups within the polymer chain and formed an ester bond [84]. The properties of prepared HGs were demonstrated by performing different tests for applicability reasons.

**Figure 9:**
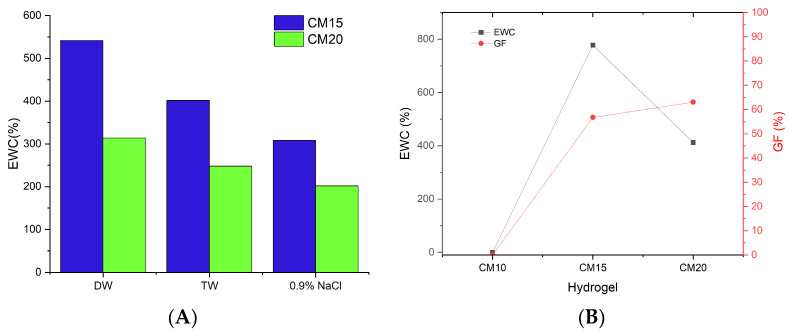
EWC% in different mediums before purification (**A**). GF% and EWC% of purified hydrogels in deionized water (**B**).

EWC% illustrates the hydrogels’ water absorption capacity, which is a key characteristic of designed hydrogels for agricultural applications [85]. It is mostly influenced by the polymer’s nature; crosslinking ratio; and medium conditions like ionic strength, pH, temperature, etc. [86]. Three different mediums (deionized water (DW), tap water (TW), and 0.9% NaCl solution) were chosen to assess the EWC% in different water types. Additionally, the tested samples in deionized water were washed and tested again in deionized water, where it was found that CM10 was semi-soluble and was thus excluded from the following tests. Figure 9A shows the effect of each medium on EWC%.

Ionic strength considerably impacts EWC% where the increment of ionic strength reduces the swelling capability [84]. However, for CM15, the EWC% was found to be 541.7%, 401.7%, and 308.5% in DW, TW, and 0.9% NaCl solution, respectively. For CM20, 313.5%, 247.7%, and 201.56% were found in DW, TW, and 0.9% NaCl solution, respectively. The electrical conductivity (EC) of the three mediums were 5 µS/cm for DW, 230 µS/cm for TW, and 15,400 µS/cm for 0.9% NaCl. Meanwhile, the increase in salt concentration resulted in an increase in EC and ionic strength, with a good correlation [87]. In addition, the purified hydrogels showed an EWC% of about 777.8% and 411.5% in DW for CM15 and CM20, respectively, as presented in Figure 9B. These findings clarify the effect of each medium on the HG swelling capacity with respect to the crosslinker concentration. These findings are relative to the findings in other studies, where Demitri et al. [84] reported that 24 h citric acid-crosslinked CMCNa, with 10% and 20% CA ratios, absorb about 500% and 300% of their weights, respectively. Capanema et al. [88] reported that prepared hydrogels from low and high molecular weight CMC absorbed about 500% when crosslinked by 10% CA and decreased as the CA ratio increased, as found in our study. In addition, different studies have shown that swelling decreased as the ionic strength increased [85,89]. Remarkably, CM20 seems to have a minor sensitivity to ionic strength compared to CM15, where a drastic decrease in EWC% was observed when the ionic strength increased.

The gel fraction (GF%) represents an important characteristic of hydrogels, determining the weight of insoluble (crosslinked) and soluble fractions, the total weight of the used polymers, and the crosslinker; the test was carried out in deionized water. Both CM15 and CM20 were stable in deionized water, where the GF% averages for CM15 and CM20 were 56.8% and 63.0%, respectively, as shown in Figure 9B. These results are similar to Saputra et al.’s findings, where 15% citric acid-crosslinked CMC had a GF% of 58% [90]. Meanwhile, Capanema et al. claimed that the GF% for CMC hydrogels with Mw 250 and 700 kDa were 60% and 80%, respectively, when 25% citric acid was used for crosslinking [88]. In addition, Durpekova et al. reported that the GF% for CMC hydrogels prepared in acid whey solutions exhibited 67.65% GF% for 15% citric acid-crosslinked hydrogel with a decreasing GF% with a decrease in citric acid concentration [85]. It is well known that there is a proportional relation between GF% and crosslinker concentration, in this case, CA [85]. These results could be attributed to the fact that when CMC is used alone, the electrostatic repulsion between the functional groups will lead to poor crosslinking. In addition, as the DS increases, the crosslinking decreases [84].

FTIR spectrometry, TGA, DTG, and microscope imaging confirmed the formation of hydrogels and their characteristics.

**Figure 10:**
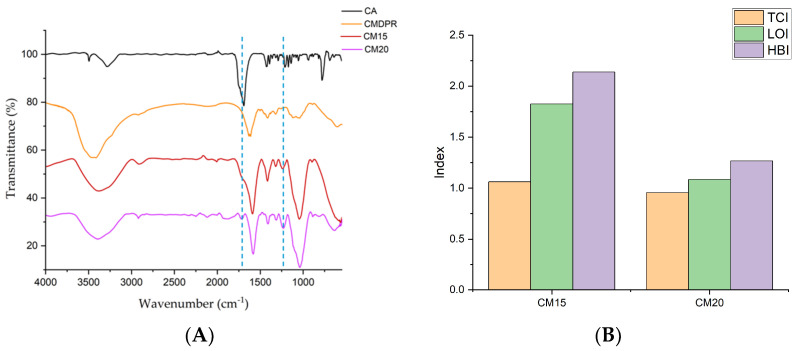
FTIR spectrum (**A**) of CA, CMDPR, CM15, and CM20 and crystallinity indices (**B**) of HGs.

FTIR spectra of hydrogels, CMDPR, and CA are depicted in Figure 10. The spectra of hydrogels showed two new peaks in the ester group compared with CMDPR of around 1235 cm^−1^ and 1726 cm^−1^. Meanwhile, the crosslinking reaction of CA with CMDPR yielded two ester bonds between the polymer chain and CA [84]. The peak intensity around 1726 cm^−1^ at CM15 showed a weak and overlapped peak, which may be attributed to poor crosslinking of CM15; a similar band was also noticed by de Lima et al. for 3% citric acid-crosslinked CMC hydrogels [91]. Also, in both samples, a weaker band at 3400 cm^−1^ was observed, which was also noticed by Capanema et al. [88]. A shift in the carboxylic band from 1618 cm^−1^ to 1587 cm^−1^ was noticed, as reported by Priya et al. [92]. In addition, TCI, LOI, and HBI indices were calculated to assess the crystallinity changes. Both CM15 and CM20 showed increased TCIs of 1.062 and 0.956, respectively. Also, an increase was noticed in LOI from 0.977 in CMDPR to 1.825 and 1.084 in CM20 and CM15, while a reduction in HBI was recorded in both samples from 3.413 to 2.138 and 1.267, respectively. The reduction in HBI is attributed to the fact that −OH groups in crosslinked hydrogel were reduced due to the esterification reaction, while the increase in both TCI and LOI may be related to the overlapping intensities of CH groups from CA and CMDPR in the region of 1425–1300 cm^−1^.

**Figure 11:**
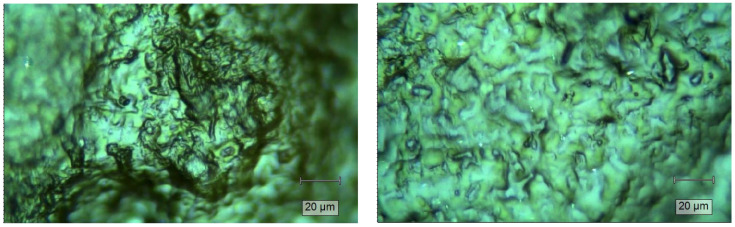
Digital microscope imaging of CM20 (**left**) and CM15 (**right**).

The morphological characteristics were recorded using microscope imaging, as shown in Figure 11. The hydrogels showed rough and rigid morphological characteristics. Also, CM20 showed a stiffer structure than CM15, most likely due to the higher crosslinker ratio. Small crystals were noticed in CM15, which were attributed to the unreacted CA, also observed through TGA. Both samples showed gaps and cavities, promoting the absorbance of high amounts of water, whereas CM20, with a higher CA ratio, showed higher porosity.

This case was in harmony with Ghilan et al.’s study, where they found that increasing the phytic acid ratio in CMC/phytic acid hydrogels increased the porosity [93]. In contrast, Pratinthong et al. reported that in 2–10% crosslinked citric acid-CMC/Poly(vinyl alcohol) hydrogels, an increase in the surface roughness and porosity were noticed from 2–6%; with increasing citric acid concentration, the pores of hydrogels were smaller [94]. In addition, Durpekova et al. mentioned that increasing citric acid concentration from 5% to 15% showed a less sponge-like structure [85]. This may be attributed to the hydrogel compositions, wherein the case of CM15 and CM20 contained residual lignin and hemicellulose, which may justify the porosity increase in respect to the crosslinker.

**Figure 12:**
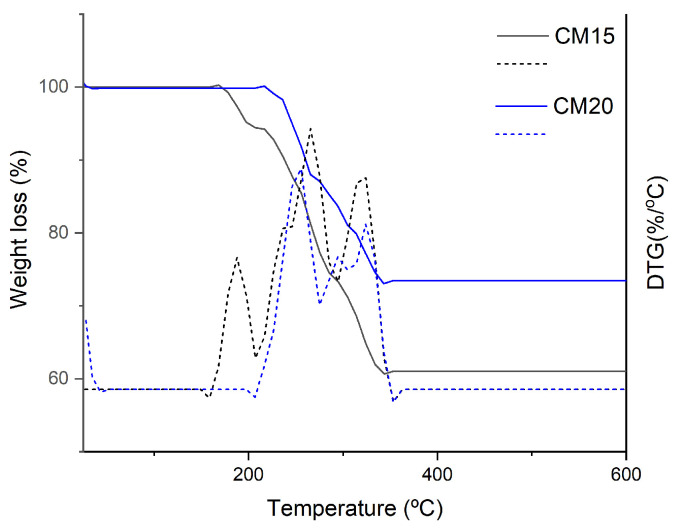
TGA and DTG analysis of CM15 and CM20.

Figure 12 presents the TG curves and DTG of the HGs. The DTG analysis of CM15 showed three major decomposition peaks in comparison with CMDPR, which showed major decomposition between 230 and 324 °C. On the other hand, CM20 showed only two. In particular, the first decomposition of CM15 was between 158.8 °C and 207.3 °C with a weight loss of about 6%, which was attributed to non-crosslinked CA that starts degrading above 148 °C [95]. This was also observed through microscope imaging. A second peak was exhibited between 207.3 °C and 343.7 °C with three steps; the first step was primarily due to the decomposition of crosslinked CA, while the last two steps represented the synchronous decarboxylation and CMDPR chains degradation which took place in CMDPR between 230 and 324 °C [88,92]. The total weight loss was about 33%. CM20 showed better homogeneity, where no non-crosslinked CA was detected. The thermal decomposition of CM20 started from 207 °C to 343.3 °C, where three decomposition steps were also noticed, with a total weight loss of 25.9%. These results are close to the results found by Priya et al. [92]. Two main decomposition peaks were noticed for 2% citric acid-crosslinked CMC. Also, de Lima et al. reported that 3% citric acid-crosslinked CMC showed two main decompositions [91]. These findings prove the presence of non-crosslinked CA within CM15 that exhibited three decomposition peaks.

### 2.6. Hydrogels’ Effect on Germination

**Figure 13:**
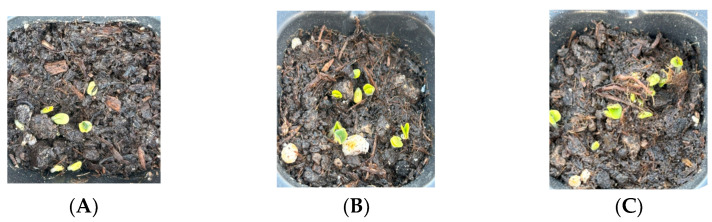

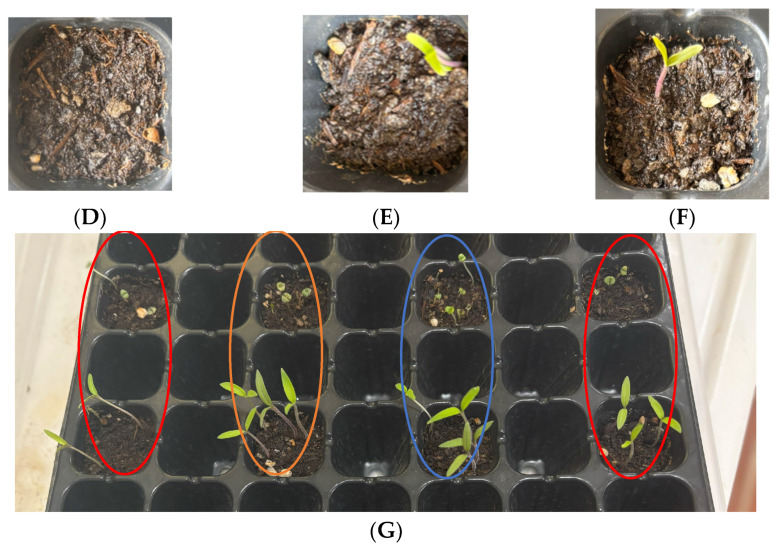
Visual images of Ch-Ref (**A**), Ch-CM15 (**B**), and Ch-CM20 (**C**) on the fourth day and To-Ref (**D**), To-CM15 (**E**), and To-CM20 (**F**) on the sixth day. Chia is in the upper row and tomato in the lower row on day 13 (**G**), where the two pots in red circles are the ref samples, the orange circle is the CM20 samples, and the blue is soil with CM15.

To assess the applicability of HGs in agricultural applications and soil water retention, the effect of the HGs on different plants’ seeds (Chico III tomato, mint, Basilico red, and chia seeds) was studied. The experiment was conducted in a seedling tray and monitored for 20 days (presented in Figure 13), with an average weather temperature of 24 degrees. The soil characteristics are presented in Table 5.

The soil was amended with 0.5% hydrogel of the soil’s total weight. Within 12 days, most seeds had already grown on different days. On the first day, the soil was irrigated and then covered with a plastic sheet for two days to prevent water evaporation. The pots were abbreviated as follows. For pots, the first two letters of the seed name were used, where “To” was for tomato, “Ch” was for chia, “Mi” was for mint, and “Ba” was for Basilico red. For example, Ch-CM15 stands for chia pot with CM15 hydrogel. “Ref” was used for soil without HGs.

## 3. Conclusions

HGs have the ability to aid the sustainability of both water and food by enhancing agriculture outcomes while reducing the amount of water needed. An eco-friendly route that reduces the cost and footprint of HG manufacturing was used for HG preparation. Date palm biomass-based carboxymethylated all-lignocellulose was crosslinked by CA to form HGs. The yielded HGs showed a positive impact on tomato, chia, mint, and Basilico red germination in comparison with reference soil. The crosslinking of CM15 and CM20 was confirmed by FTIR analysis and GF%. TGA revealed the thermal stability of HGs prepared below 200 °C. In addition, TGA revealed that in CM15, some unreacted CA was confirmed by microscopic imaging, which explains the low GF%. CM15, after purification, showed an EWC% of about 777.8%, while CM20 showed about 411.5%. Furthermore, a good EWC% was also observed in tap water and saline water (0.9%NaCl) with unpurified hydrogels.

These findings support the utilization of a modified lignocellulosic matrix for low-cost hydrogel fabrication without any fractionation, resulting in HGs with suitable properties for soil water retention and different agricultural applications; however, the effect of the all-lignocellulose composition and molecular weight, alongside HGs’ stability and performance in different weather conditions, soil types, and pH mediums, must be investigated for agricultural usage. Further, future studies should consider nutrient and fertilizer loading and release investigations.

## 4. Materials and Methods

### 4.1. Materials

Date palm rachis (DPR) were gathered from the campus of King Abdulaziz University in Jeddah City, Saudi Arabia. Ethanol and methanol were purchased from Sigma Aldrich, St. Louis, MI, USA. Isopropanol (IPA), monochloroacetic acid (MCA), and sulfuric acid (H_2_SO_4_) were purchased from BDH Chemicals Ltd., Poole, UK. Sodium hydroxide (NaOH) was purchased from Merck, Billerica, MA, USA. Citric acid was purchased from Techno Pharmchem, Bahadurgarh, India. Commercial carboxymethyl cellulose (C-CMC) with 0.7–0.8 DS was purchased from BDH Chemicals Ltd., Poole, UK, and was used for comparison purposes.

### 4.2. Biomass Compositional Analysis and Pretreatment

The collected DPR was washed with tap water and wiped with a cloth to remove any physically attached impurities, then left to dry for a week at room temperature. The dried DPR was subjected to physical pretreatment where DPR was chopped by a wood saw until it became stick-like, then grounded by a coffee grinder. In sequence, to get fine powdered biomass, the DPR was sieved by a 1 mm US standard sieve, milled in a Laboratory Mill 3100 (Perten Instruments, Huddinge, Sweden), and then the fine powder was sieved by a 0.21 mm mesh. The compositional analysis for the powdered DPR was performed according to NERL LAPs: NREL/TP-510-42622 for ash content [96], NREL/TP-510-42619 for extractives content [97], NREL/TP-510-42618 for lignin content [98], and TAPPI standard (T 203 cm-99) for cellulose and hemicellulose contents [99].

### 4.3. Biomass Carboxymethylation

In the modification for carboxymethylated date palm rachis (CMDPR) preparation, the DPR was subjected to hot water treatment, where 20 g of DPR was treated with 1 L hot water for 1.5 h at 90–100 °C to eliminate the water-soluble matters to avoid its reaction with MCA that would lead to MCA consumption. The water-treated DPR (WTDPR) was left to dry at room temperature until a constant weight was achieved. The modification reaction was established as follows: 5 g of the air-dried WTDPR was suspended in 105 mL of IPA at room temperature for 15 min. Further, 25 mL of 30% NaOH was added to the suspension portion-wise and kept under stirring for 1 h. Later, a solution of 6 g MCA dissolved in 20 mL of IPA was added to the suspension and heated at 55 °C for 3.5 h under stirring. After the end of the reaction, the mixture was left to reach room temperature and then filtered. The residual was suspended in 100 mL of absolute methanol and neutralized by 90% acetic acid. Then, it was washed four times with 70% ethanol and then with absolute ethanol. The CMDPR was left in a drying oven at 50 °C until a constant weight was achieved.

### 4.4. CMDPR Degree of Substitution and Yield

The yield and DS for the prepared CMDPR was determined using the same method for determining the degree of substitution method based on pure cellulose according to [79] with some modifications. In detail, 0.5 g of CMDPR was suspended in 20 mL of HNO_3_–methanol (1:1 *v*/*v*) solution and left undisturbed for 3 h. In sequence, the solution was rinsed with 70% methanol solution (*v*/*v*) and then dried in an oven until no weight changes were observed. Later, 0.2 g of the product was dissolved in 20 mL of deionized water and 3 mL of 1 N NaOH. This solution was titrated against 1 N HCl using phenolphthalein as an indicator. The titration was considered complete when the color transitioned from red to yellow. The DS was calculated using Equations (1) and (2) [79]. The yield of CMDPR was calculated according to Equation (3) [79].

DS = 0.162A/(1 − 0.0584A)(1)

A = (BC − DE)/F(2)

where

A is milliequivalents of acid consumed per gram of sample.
B is NaOH solution added amount in mL.
C is the concentration of the NaOH solution in normality.
D is the amount of HCl consumed in the titration in mL.
E is the concentration of the HCl in normality.
F is acidified CMDPR used (g).
The value 162 is the gram molecular mass of the anhydrous glucose unit of cellulose.
The value 584 is the net increase in molecular mass for each carboxymethyl group substituted.

Yield of CMDPR (%) = (W_p_/W_0_) × 100% (3)

where

W_p_ is the weight of produced CMDPR.
W_0_ is the weight of WTDPR.

### 4.5. Hydrogels Fabrication

For the fabrication of hydrogels, CMDPR was crosslinked by citric acid in three different ratios, which were 10%, 15%, and 20% of CMDPR weight, which corresponded to 200 mg, 300 mg, and 400 mg, respectively. Three solutions of CMDPR were prepared with 2% consistency. In detail, 2 g of CMDPR was dissolved in 100 mL overnight. Then, an adequate amount of crosslinker was added to each solution and left to dissolve for 1 h. Further, 15 mL of each solution was cast in silicon molds and left to dry at room temperature for 3 days to form hydrogel films. The dried films were cured in an oven at 80 °C for 24 h to get the crosslinked hydrogels.

### 4.6. Hydrogel Properties

#### 4.6.1. Gel Fraction

The gel fraction (GF%) represents an important characteristic of hydrogels, determining the weight of insoluble (crosslinked) and soluble fractions. To determine the gel fraction of the prepared hydrogels, a certain weight (W_0_, initial mass) of each hydrogel was immersed in 100 mL of deionized water for 24 h at room temperature, then brought out and wiped with tissue paper to remove the adsorbed water on the surface, and then its weight was recorded (W_s_, swollen mass). Later, the hydrogel was dried in an oven at 50 °C until a constant weight (W_f_, final mass) was achieved. The GF% was calculated using Equation (4) [88].

GF (%) = ((W_0_ − W_f_)/W_0_) × 100% (4)

#### 4.6.2. Equilibrium Swelling Capacity

The hydrogels’ equilibrium swelling capacity (EWC%) was examined in three different media and determined gravimetrically. A known hydrogel weight was taken and then soaked in 100 mL of the examination solution: DW, TW, and 0.9% NaCl, with EC of 5 µS/cm, 230 µS/cm, and 15,400 µS/cm, respectively. The hydrogel was kept in the solution for equilibrium for 24 h at room temperature. After that, the swollen hydrogel was carefully removed from the solution using tweezers; the surface water was wiped with tissue paper; and then the hydrogel was weighed to get the EWC%, which was determined according to Equation (5) [88].

EWC (%) = ((W_S_ − W_0_)/W_0_) × 100% (5)

### 4.7. Effects of Hydrogels on Germination

Four different plant seeds were tested in a seedling tray to study the effect of the prepared hydrogels on germination, which were abbreviated using the first two letters of the seed: “To” for tomato, “Ch” for chia, “Mi” for mint, and “Ba” for Basilico red. The soil used in this study was commercial organic soil with a pH range of 6–7, EC 1.78 µS/cm, 2.08% nitrogen content (N), 0.015% phosphorous content (P), and 0.22% potassium content (K). The tray was divided into 4 groups, each group consisting of 4 cavities, for a total of 16 cavities. Two groups were defined as references with unmodified soil, which were abbreviated as “Ref”, and two groups with hydrogel-amended soil using CM15 and CM20. For the hydrogel-amended groups, the hydrogels were grounded and mixed thoroughly with the soil, where the percentage of hydrogel was 0.5% of the soil weight. Each cavity was filled with approximately 8 g of soil.

### 4.8. Characterization

#### 4.8.1. Fourier Transform Infrared Spectrometry (FTIR)

The samples were recorded through a Fourier transform infrared spectroscope to investigate the structural changes and crystallinity indices. Powder samples were recorded using the KBr pellet method using Nicolet iS50 FT–IR (Thermo Scientific, Madison, WI, USA) with a resolution of 4 cm^−1^ for 32 scans. CA and prepared HGs were recorded with a Fourier-transform Infrared Spectrometer in attenuated total reflection (ATR) mode using Spectrum 100, (Perkin Elmer, Shelton, CT, USA) with a 4 cm^−1^ resolution for 16 scans. The scanning range was from 500 cm to 4000 cm^−1^ for all samples. Crystallinity indexes of samples were evaluated using the lateral order index (LOI, A1425/A897), which is the ratio between the absorbance of CH_2_ around 1430–1420 cm^−1^ and the absorbance of β-glycosidic bonds around 900–890 cm^−1^ [55,56]; total crystallinity index (TCI, A1374/A2919), which is the ratio of between the absorbance of –CH stretching vibration around 2900 cm^−1^ and the absorbance of CH deformation vibration around 1374 cm^−1^ [55,57]; and hydrogen bond intensity (HBI, A3336/A1321), which is the ratio between the absorbance of OH stretching and hydrogen bonding between molecules around 3330–3400 cm^−1^ and the absorbance of ~1320 cm^−1^ from CH rocking vibration of the glucose ring [58,59]. To calculate the crystallinity indexes, the transmittance values (T) were converted to absorbance (A) using Equation (6) from Beer’s Law [100].

A = 2 − log_10_ T (6)

#### 4.8.2. Microscope Imaging

The morphological properties of the sample were recorded using a research-grade digital microscope with a 50× magnification and 50× objective lens on a Leica DM2700 optical microscope (Wetzlar, Germany). The sample images were directly taken in a dry state without any modification.

#### 4.8.3. Dynamic Light Scattering (DLS)

Dynamic light scattering analysis was performed to investigate the particle size distributions using a Mastersizer 3000 equipped with an Aero S dispersion unit (Malvern Instruments Ltd., Malvern, UK). The air pressure was set to 2 bar with a 20% feed rate. For the analysis, 0.25 g of a sample was used.

#### 4.8.4. Thermal Analysis

Thermal behaviors were investigated by Thermogravimetric Analysis (TGA) using a Synchronous Thermal Analyzer STA-1200 (Bioevopeak, Jinan, China). The heating rate was 10 °C/min from 30 °C to 600 °C. Also, the samples’ behaviors were studied using differential scanning calorimetry (DSC) using DSC 823 (Mettler Toledo, Nänikon, Switzerland) from 30 °C to 300 °C with a heating rate of 5 °C/min.

## Figures and Tables

**Table 1:** Chemical composition of date palm rachis from different countries and locations.

Biomass Type	Cellulose	Hemicellulose	Lignin	Ash	Extractives	Hot Water Soluble	Ref.
Date palm rachis from Jeddah City in Saudi Arabia	29.82	21.69	13.74	5.89	9.5	10.84	This work
Date palm rachis from Marrakesh City in Morocco	39.8	31.4	14.0	9.2	NA *	16.8	[31]
Date palm rachis from southern Algeria	35.87	NA *	16.94	NA *	8.66	NA *	[37]
Date palm rachis from Biskra City in Algeria	47.31	25.72	15.67	5.47	5.80	NA *	[35]
Date palm rachis from Khoozestan City in Iran	38.26	28.17	22.53	5.96	5.08	NA *	[36]

* NA: not available or mentioned.

**Table 2:** Comparison of FTIR spectra of samples.

Peak Assignment	Peak Wavenumber (cm^−1^)						Ref.
−OH _stretching_	**DPR**	**WTDPR**	**CMDPR**	**C-CMC**	**CM15**	**CM20**	
−CH _stretching_	3400	3400	3400	3400	3384	3402	[41]
C=O _ester or −COOH_	2919	2919	2919	2919	2913	2923	[41]
C=O _conjugated_	1731	1731	-	-	1726	1726	[42,43]
C=C/C=O _aromatic or carboxylic acid_	1637	1637	1637	-	-	-	[43]
C=C _aromatic_	1618	1618	1618	1618	1587	1586	[44,45]
−CH _aromatic_	1507	1507	1507	-	-	-	[46,47]
−CH_2 aliphatic_/−CH _aromatic_	1458	1458	1458	-	-	-	[46,47]
−CH _deformation_	1425	1425	1422	1420	1414	1414	[47]
−CH _rocking_	1374	1374	1374	-	-	-	[47]
C−O _aromatic_	1321	1321	1330	1330	1318	1316	[47]
C−O−C _stretching_	1250	1250	1270	-	1233	1235	[48]
C−C/C−O	1158	1158	1158	1158	-	-	[46,47]
C−O _stretching_	1108	1108	1120	1120	-	-	[49]
C−O−C _β-glycosidic_	1049	1049	1049	1059	1042	1042	[46,47]
−OH _stretching_	897	897	897	901	898	898	[50]

**Table 3:** Comparison of different studies on carboxymethylation of lignocellulosic matrix.

Starting Material	Conditions	DS	Ref.
Date palm rachis	30% NaOH, 1 g substrate/1.2 g MCA, 55 °C, 3.5 h, Isopropanol	1.14	This work
Date palm rachis	40% NaOH, 1 g substrate/1.749 g MCA, 80 °C, 8 h in n-Butanol	1.17	[68]
Posidonia oceanica	40% NaOH, 1 g substrate/1.749 g MCA, 80 °C, 8 h in n-Butanol	1	[68]
Cunninghamia lanceolata	30% NaOH, 1 g substrate/1.4 g MCA, 80 °C, 2 h, Isopropanol	1.36	[69]
Lepidium Polyuronide	45% NaOH, 1 g substrate/15 g MCA, 70 °C, 2 h	1.75	[70]

**Table 4:** Summary of TGA analysis results.

Sample	Stage	Range	Decomposition Assignment	Residue (%)
CMDPR	1st	50–180 °C	Volatile compounds and moisture	45.0%
	2nd	230–324 °C	Carboxyl and hydroxyl groups	
	3rd	324–373 °C	Cellulose chain	
	4th	382–421 °C	Methyl-aryl ether bonds of lignin	
	5th	421–460 °C	Lignin chain	
	6th	590–600 °C	Oxidation of residual carbonaceous	
C-CMC	1st	50–180 °C	Volatile compounds and moisture	23.8%
	2nd	246–314 °C	Carboxyl and hydroxyl groups	
	3rd	360–400 °C	Cellulose chain	
	4th	587.5–600 °C	Oxidation of residual carbonaceous	
CM15	1st	158.8–207.3 °C	Non-crosslinked CA	67.0%
	2nd	207.3–343.7 °C	Crosslinked CA and CMDPR chain	
CM20	1st	207–343.3 °C	Crosslinked CA and CMDPR chain	74.1%

**Table 5:** Used soil characteristics.

pH	EC	N	P	K
6–7	1.78 µS/cm	2.08%	0.015%	0.22%

## Data Availability

Data are contained within the article.
